# Publisher Correction: Using survey data to estimate the impact of the omicron variant on vaccine efficacy against COVID‑19 infection

**DOI:** 10.1038/s41598-023-28948-8

**Published:** 2023-01-31

**Authors:** Jesús Rufino, Carlos Baquero, Davide Frey, Christin A. Glorioso, Antonio Ortega, Nina Reščič, Julian Charles Roberts, Rosa E. Lillo, Raquel Menezes, Jaya Prakash Champati, Antonio Fernández Anta

**Affiliations:** 1 CoronaSurveys Team, Madrid, Spain; 2 IMDEA Networks Institute, Av. Mar Mediterráneo 22, 28918 Leganés, Madrid, Spain; 3 University of Porto & INESC TEC, Porto, Portugal; 4 Univ Rennes, IRISA, CNRS, Inria, 35042 Rennes, France; 5 Academics for the Future of Science, Inc. & University of California San Francisco, San Francisco, USA; 6 University of Southern California, Los Angeles, USA; 7 Jožef Stefan Institute, Department of Intelligent Systems, Ljubljana, Slovenia; 8 Skyhaven Media, Liverpool, UK; 9 University of Carlos III de Madrid, Getafe, Madrid, Spain; 10 Centre of Mathematics of University of Minho, Braga, Portugal

Correction to: *Scientific Reports* https://doi.org/10.1038/s41598-023-27951-3, published online 17 January 2023

The original version of this Article contained a spelling error in the name of the author Nina Reščič, which was incorrectly given as Nina reščič.

The original Article has been corrected.

